# R Loops in the Regulation of Antibody Gene Diversification

**DOI:** 10.3390/genes8060154

**Published:** 2017-06-02

**Authors:** Rushad Pavri

**Affiliations:** Research Institute of Molecular Pathology (IMP), Campus Vienna Biocenter-1, Vienna Biocenter, Vienna 1030, Austria; rushad.pavri@imp.ac.at

**Keywords:** class switch recombination (CSR), activation induced deaminase (AID), R loops, Q-quadruplexes (G4)

## Abstract

For nearly three decades, R loops have been closely linked with class switch recombination (CSR), the process that generates antibody isotypes and that occurs via a complex cascade initiated by transcription-coupled mutagenesis in switch recombination sequences. R loops form during transcription of switch recombination sequences in vitro and in vivo, and there is solid evidence that R loops are required for efficient class switching. The classical model of R loops posits that they boost mutation rates by generating stable and long tracts of single-stranded DNA that serve as the substrate for activation induced deaminase (AID), the enzyme that initiates the CSR reaction cascade by co-transcriptionally mutating ssDNA in switch recombination sequences. Though logical and compelling, this model has not been supported by in vivo evidence. Indeed, several reports suggest that R loops may not be involved in recruiting AID activity to switch regions, meaning that R loops probably serve other unanticipated roles in CSR. Here, I review the key findings in this field to date and propose hypotheses that could help towards elucidating the precise function of R loops in CSR.

## 1. Introduction

R loops are structures that form when nascent RNA stably hybridizes to its template DNA resulting in the non-template DNA strand being extruded as long, stable single-stranded DNA (ssDNA). In recent years, there have been a slew of publications demonstrating the widespread genomic presence of R loops with implications in various biological processes [1]. For example, R loops have been implicated in recruitment of chromatin regulators [2], heterochromatin formation [3], and transcription termination [4,5]. Importantly, an excess of R loops has been associated with increased formation of DNA breaks, replication fork stalling, and collisions between transcription and replication complexes, all of which can lead to genome instability [6,7]. To counter such destabilization, there are several proteins that have been found to prevent R loop formation or eliminate R loops rapidly, such as Senataxin [8], Xrn2 [9], TDRD3/TOP3b complex [10], BRCA2 [11], and the exosome complex [12,13]. Indeed, mutations of R loop-suppressing factors are observed in some cancers and neurological diseases and correlate with increased R loop formation [14].

**Figure 1:**
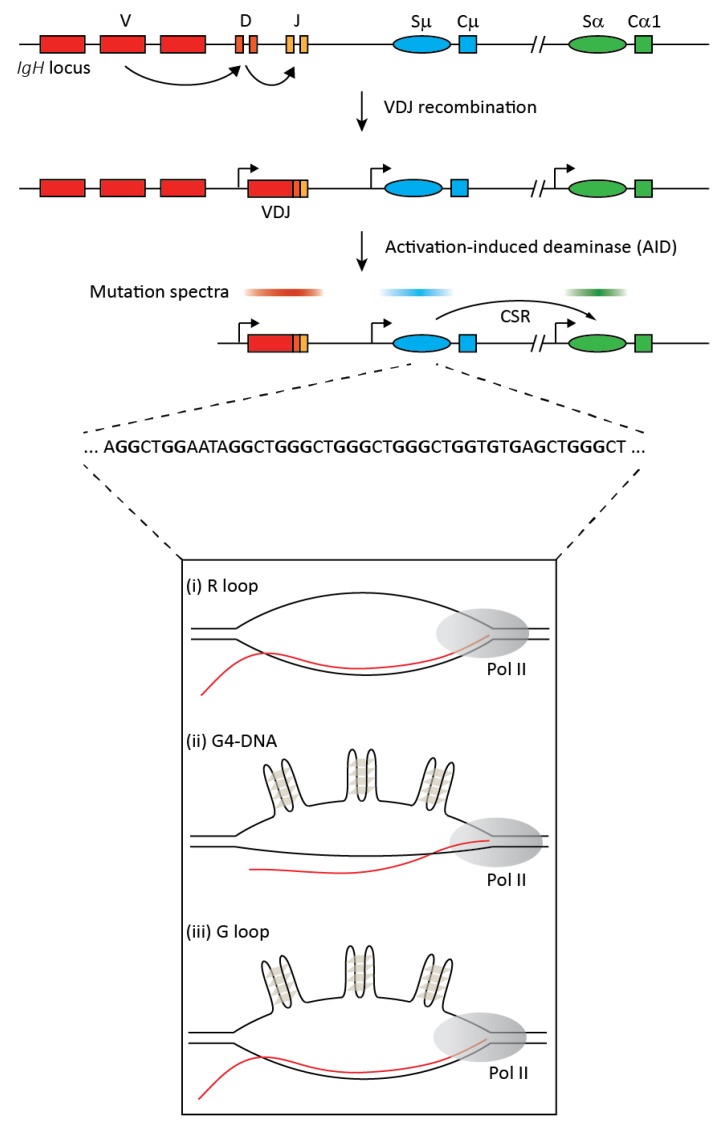
The upper part of the figure provides a simplified overview of class switch recombination (CSR) at the immunoglobulin heavy chain (*IgH*) locus. The germline locus comprises multiple variable (V), diversity (D), and junctional (J) exons. Each antibody isotype has a unique constant (C) region exon that is preceded by a repetitive sequence called the switch (S) region and a promoter that generates non-coding transcripts. Random recombination between V, D, and J exons via VDJ recombination results in a functional heavy chain of the IgM isotype with clonal diversification of the antibody repertoire. B cell activation upon antigen encounter leads to expression of the activation induced deaminase (AID), which generates mutations in ssDNA in co-transcriptional manner mostly in the VDJ and switch regions (indicated by the mutation spectra). Mutations in switch regions are processed to DNA breaks that serve as substrates for the non-homologous deletional recombination between the donor (Sμ) and an acceptor (Sα in this example) switch region leading to CSR and the expression of a new antibody isotype, IgA. The repetitive switch regions range from 2 kb to 12 kb, and switch sequences have high G clustering and high overall G-richness on the non-template strand (a representative stretch of switch region sequence is depicted). Upon transcription, these G-rich regions can form secondary structures like R loops, G-quadruplexes (G4), and G-loops, as shown in the lower panel.

Although these recent findings have reignited research into R loops, this review will focus on our current understanding of R loop biology in the context of antibody diversification at the immunoglobulin heavy chain (*IgH*) locus in B lymphocytes, where a physiological role for R loops was proposed nearly three decades ago. Specifically, R loops have been linked to the process of class switch recombination (CSR), which results in the production of the various antibody isotypes that serve crucial effector functions during the humoral immune response [15,16] (Figure 1). CSR is initiated by the mutator enzyme, activation induced deaminase (AID) [17,18], which has a high density of hotpot motifs in the G-rich, repetitive switch sequences of the *IgH* locus (Figure 1). Upon transcription, these switch sequences form R loops, and AID was found to act co-transcriptionally on ssDNA [19,20,21,22,23,24]. This gave rise to a model wherein R loops were proposed to enhance AID activity by providing it with abundant ssDNA substrate [19]. Yet, as I discuss below, this model is not supported by empirical evidence, and there remain lingering issues regarding the role of R loops in AID biology and CSR.

## 2. Transcription in CSR and SHM

The first indication that CSR was linked to transcription came from the observation that CSR was accompanied by the appearance of non-coding transcripts from the germline promoters of the corresponding switch regions [25,26]. These findings were confirmed and extended by several subsequent studies that led to the conclusion that CSR was preceded by germline transcription through the switch and constant regions [27,28]. In addition, a host of studies had already led to the important conclusion that transcription was critical for mutation of both *IgH* variable and switch regions during antibody diversification (reviewed in [27,29,30]). This naturally led the field to investigate the role of transcription in CSR and whether these non-coding switch region transcripts were involved in mutagenesis or some other aspect of the CSR mechanism.

## 3. Discovery of R Loops in *IgH* Switch Regions

Initial evidence that transcribed *IgH* switch regions generate R loops came from in vitro transcription experiments where RNA/DNA hybrids were detected during transcription of *IgH* switch α (Sα) sequences by bacterial polymerases, suggesting that the transcribed RNA was stably bound to the template DNA [31]. The most striking observation herein was that these unusual structures were observed only when the switch sequence was transcribed in its physiological orientation, thus clearly implicating sequence composition as an important determinant in the formation of R loops [31]. Similar conclusions regarding the formation and orientation-dependence of R loops in switch regions were reached via in vitro transcription of Sμ, Sγ2b, and Sγ3 sequences [32]. Although the biology of R loops was poorly understood at the time and AID had not yet been discovered, these findings nonetheless provided a key insight into the transcriptional landscape of switch regions and suggested that switch region sequence composition could be of functional relevance in CSR.

The crucial in vivo evidence for the existence of R loops came from the work of Lieber and coworkers in 2003 who used sodium bisulfite modification followed by DNA sequencing to demonstrate the presence of long ssDNA stretches in the Sγ3 and Sγ2b regions of primary murine B cells stimulated to undergo CSR to IgG3 and IgG2b [33]. The ssDNA stretches were detected almost exclusively on the non-template G-rich strand of the switch regions and reached >1 kb in length, which clearly implied the presence of R loops in these loci [33]. These data firmly established the idea that R loops are stable intermediates of CSR that arise via the association of the switch region DNA and its cognate, nascent RNA, a result that has recently been corroborated using transgenic mice over-expressing RNaseH, an enzyme that specifically degrades RNA within RNA/DNA hybrids [34]. In addition, *Xenopus* switch regions, which lack the extensive G-richness of mammalian switch regions and were therefore predicted not to form R loops, have in fact been found to harbor R loops [35] likely because they possess GG dinucleotide motifs which appear sufficient for R loop initiation [36,37].

## 4. R Loop Frequency Correlates with CSR Efficiency

Early support for a functional role of R loops in CSR came from Alt and coworkers who demonstrated that inverting the 12 kb switch γ1 (Sγ1) sequence in mice (which would be unable to form R loops) led to significantly decreased CSR to IgG1. Furthermore, replacement of Sγ1 with an artificial 1 kb G-rich cassette capable of R loop formation could partially rescue CSR, whereas the inverted sequence could not [38]. Subsequently, in vitro transcription assays using T7 polymerase revealed that dsDNA templates that could form R loops were significantly better substrates for AID than those that could not [19,39]. More recently, the Lieber group performed an elegant and systematic investigation into the role of R loops in CSR in which *IgH* Sαsequences in the murine B cell line, CH12, were replaced with artificial sequences having varying strengths of R loop formation and AID hotspot densities. Importantly, they found that a high density of AID hotspots in the absence of R loop formation results in inefficient CSR, but that a combination of AID hotspot density and R loop strength gave the highest CSR efficiency [40]. Altogether, these studies strongly suggest that R loops have a physiological role in CSR.

## 5. Are R Loops Required for *IgH* Switch Region Mutagenesis? Probably Not

The discovery of ssDNA as the substrate for AID led to the model that the ssDNA tracts made available by the R loop would provide a stable substrate for AID, leading to higher rates of mutation that, in turn, would enhance CSR efficiency. However, direct evidence for this idea is still lacking. In the Zhang et al. study just discussed [40], AID recruitment and mutation analyses were not performed, so we do not know whether the observed correlation between CSR and R loop strength was directly related to increased AID recruitment, mutation frequency, or some other mechanism.

To address the role of R loops in antibody diversification, perturbation of R loop frequency by over-expression of RNaseH, which recognizes RNA/DNA hybrids, has been frequently employed followed by detection with the convenient DRIP assay that utilizes the S9.6 antibody to immunoprecipitate RNA/DNA hybrids [41]. Using this approach, studies in the CH12 B cell line did not report any effect on switch region mutation frequency following RNaseH over-expression [42] or via short-term retroviral expression in activated B cells [43,44]. However, whereas one study did not report CSR defects in RNaseH-expressing stable CH12 lines [42], another study reported ~50% decrease in CSR following retroviral infection of RNaseH [43]. Finally, RNaseH over-expression in transgenic mice resulted in a slight increase in mutation on the template strand but did not alter CSR frequency [34].

In addition to switch regions, *Ig* variable regions are also potent targets of AID resulting in somatic hypermutation and diversification of antigen-binding [30,45]. However, variable genes are not G-rich and are not predicted to form R loops. Indeed, studies in hypermutating human Ramos cells did not report R loop formation in the *IgH* variable region [46] and RNaseH expression did not alter the rates of variable gene somatic hypermutation [42]. In contrast, the avian B cell line, DT40, which undergoes AID-mediated gene conversion, was found to harbor R loops in the variable region of the *IgK* gene; however, mutation rates were unaffected by RNaseH-mediated R loop depletion [47]. In all these studies, the authors concluded that R loops were not required for AID targeting and activity.

It must be noted, however, that there are two caveats regarding the interpretation of the RNaseH over-expression and DRIP studies. Firstly, R loops are not completely abolished under these conditions. In fact, even in RNaseH-expressing transgenic B cells, where RNaseH mRNA levels were measured to be >100-fold higher than wild-type, R loop frequency was reduced by 70% in the *IgH* switch μ (Sμ) locus and by just 50% in the β-actin gene [34]. Secondly, the DRIP assay measures only the relative steady-state presence of R loops in a bulk population setting but does not provide information on the length of individual R loops or the kinetics of R loop formation; indeed, the assay readout is likely a combination of all these factors. Consequently, one cannot conclude whether the reduced DRIP signal upon RNaseH expression reflects reduced R loop formation, decreased R loop length, more transient R loop formation, or some combination of these. An additional detection approach such as the sodium bisulfite-based assay [33], which also allows R loop tract length determination, could be performed to gain better insights into the nature of R loop perturbation with RNaseH. Due to these limitations, one can conclude from these studies that R loop frequency is clearly not correlated with mutation rates, but one cannot conclude that R loops play no role in AID targeting.

That said, a recent study from the Alt group, using an entirely different approach, lends strong support to the idea that R loops are not involved in AID targeting. They generated transgenic mice wherein the *IgH* variable region was replaced with a core switch region cassette in the physiological or non-physiological orientation. In either orientation, they observed equally high rates of mutation and a similar mutation signature [48]. These results lead to three conclusions: (1) The mutagenesis step of CSR is independent of switch region orientation. (2) Since R loops form efficiently in the physiological orientation but not in the non-physiological one [31,32], R loops are probably not contributing to AID activity. (3) Finally, since non-physiologically oriented switch regions are significantly impaired in supporting CSR [38], the orientation-independence of AID activity strongly suggests that R loops play some other role in CSR independent of AID activity.

## 6. R Loops Influence CSR by Regulating the DNA Replication Landscape at the *IgH* Locus

In line with alternative roles for R loops in CSR, a recent study has implicated R loops in promoting the long-range synapsis of donor and acceptor switch regions that is essential for the final, end-joining phase of CSR [43]. Specifically, it was reported that CSR efficiency is dependent on, and correlates with, the activity of origins of replication in switch regions and that R loops contribute to the specification of these origins during early S phase [43]. Indeed, defects in origins establishment were observed upon shRNA-based depletion of subunits of the replicative helicase, the Mcm complex, as well as upon depletion of R loops with RNaseH. Furthermore, both perturbations led to impaired long-range switch region synapsis and a CSR defect independent of AID activity [43]. This finding shows for the first time that switch region R loops have an additional function in CSR at a step downstream of mutagenesis. Intriguingly, this suggests that the non-physiologically oriented switch regions, which have not been shown to harbor R loops [31,32] and poorly support CSR [38] despite normal targeting of AID activity [48], may be impaired in the firing of origins of replication and switch region synapsis. A postulate from all these studies is that increased R loop strength would lead to increased origin activation. This hypothesis can be tested using switch region replacement systems where R loop strength was shown to correlate well with CSR efficiency [37].

## 7. G-Quadruplexes in *IgH* Switch DNA and Their Functional Implications vis-à-vis R Loops

It is important to note that in the context of mammalian switch regions, the G-rich non-template ssDNA and the corresponding nascent RNA can also form G-quadruplexes (G4) [49,50] (Figure 1). Indeed, G4 structures were proposed to form in *IgH* switch regions almost three decades ago by Sen and Gilbert [51]. G4 are four-stranded structures formed via Hoogstein base-pairing between guanines forming a planar quartet (G-quartet), which can stack with adjacent such planes [52] (Figure 1). In addition, in vitro transcription of switch regions revealed the formation of G-loops, which result when the non-template ssDNA, released due to base-pairing between the nascent RNA and template DNA strand, folds into a G4 configuration [49] (Figure 1).

The presence of G4 structures in switch region DNA and RNA has two implications for CSR. Firstly, G4 DNA has been strongly correlated with active origins of replication in multiple genome-wide studies [53,54,55,56] and, in vitro, the origin recognition complex (ORC) binds efficiently to G4-containing ssDNA and RNA, but poorly to dsDNA [57]. Thus, G4 DNA density may contribute to CSR efficiency, in part, by increasing the frequency of origins of replication, thereby enhancing long-range synapsis between recombining switch regions [43]. It is unknown whether G4 DNA can form independently of R loops in switch regions or whether stable R loop formation is required to stabilize G4 DNA. Thus, it will be important to determine what effect RNaseH treatment has on G4 DNA in switch regions.

Secondly, spliced, intronic *IgH* switch transcripts that form G4 RNA structures can bind AID and target it to switch regions for mutation. Indeed, an AID mutant (G133V) that fails to bind G4 RNA is unable to support efficient CSR despite having normal enzymatic activity [50]. Since the AID-G4 RNA association is a post-splicing event, this mode of AID targeting appears to be independent of R loops. In light of this, an important question that arises is whether G4-rich sequences are sufficient for the AID targeting and mutation steps of CSR, and whether R loops play any role in this process. By uncoupling G4 formation from R loop formation, for instance, by replacing switch regions with sequences that form G4 structures but not R loops, and vice versa, one could separately determine the contribution of each structure towards AID targeting, mutation, and replication origin specification during CSR.

## 8. Conclusions

After nearly three decades of research, we have learned that R loops are stable intermediates of CSR and, more importantly, that R loop frequency correlates with the efficiency of CSR. Thus, we can conclude that R loops must play an important role in CSR. However, in contrast to the original model, multiple and diverse studies have consistently demonstrated that R loops are unlikely to play a role in the initial AID-dependent mutagenesis phase of CSR and SHM. In the context of CSR, the major conclusion from all these efforts is that R loops must have other AID-independent roles in CSR, for example, in switch region synapsis as shown recently, and future research should be aimed at elucidating such AID-independent roles of R loops. Along these lines, the role of G4 DNA in switch regions has barely been explored and merits serious investigation since it is plausible that these structures are functionally relevant for CSR in the context of stable R loop formation.

## Supplementary Materials

Supplementary File 1
