# Supplementary material for: R Loops in the Regulation of Antibody Gene Diversification
Source: Genes (Basel). 2017 Jun 2;8(6):154. doi: 10.3390/genes8060154 (PMC5485518; doi:10.3390/genes8060154)
Supplement: Supplementary File 1 [file genes-08-00154-s001.pdf]

Figure Legend

The upper part of the figure provides a simplified overview of class switch recombination (CSR) at the immunoglobulin heavy chain (*IgH*) locus. The germline locus comprises multiple variable (V), diversity (D) and junctional (J) exons. Each antibody isotype has a unique constant (C) region exon that is preceded by a repetitive sequence called the switch (S) region and a promoter that generates non-coding transcripts. Random recombination between V, D and J exons via VDJ recombination results in a functional heavy chain of the IgM isotype with clonal diversification of the antibody repertoire. B cell activation upon antigen encounter leads to expression of the activation induced deaminase (AID) which generates mutations in ssDNA in co-transcriptional manner mostly in the VDJ and switch regions (indicated by the mutation spectra). Mutations in switch regions are processed to DNA breaks that serve as substrates for the non-homologous deletional recombination between the donor (Sμ) and an acceptor (Sα, in this example) switch regions leading to CSR and expression of a new antibody isotype, IgA. The repetitive switch regions range from 2-12 kb and switch sequences have high G clustering and high overall G-richness on the non-template strand (a representative stretch of switch region sequence is depicted). Upon transcription, these G-rich regions can form secondary structures like R loops, G-quadruplexes (G4) and G-loops, as shown in the lower panel.
